# Supplementary figures and images for: Avidity sequencing of whole genomes from retinal degeneration pedigrees identifies causal variants
Source: PLoS One. 2024 Oct 4;19(10):e0307266. doi: 10.1371/journal.pone.0307266 (PMC11457586; doi:10.1371/journal.pone.0307266)

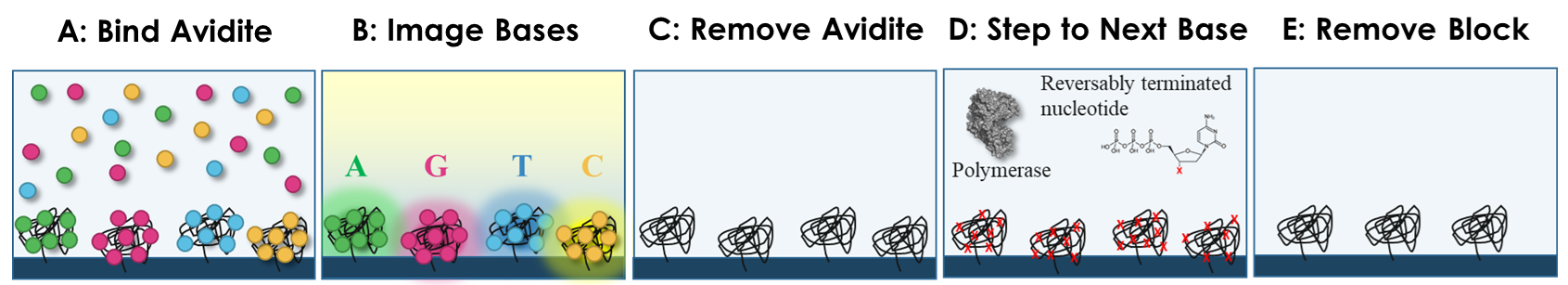

Supplement: S1 Fig — The number of sequencing cycles equals the total read length. The figure depicts four DNA concatemers attached to the flowcell surface. In the illustrated cycle, each of the four are being sequenced at different nucleotides, so bind each of the four avidites (A). The imaging step (B) determines the base, followed by avidite removal (C). An unlabeled nucleotide is incorporated (D) to advance along the template strand and the block is removed (E) so that the next cycle of sequencing can start. (TIF) [file pone.0307266.s001.tif]
